# Phytobezoar Impaction Within Meckel's Diverticulum Causing Distal Small Bowel Obstruction: A Case Report With Characteristic Computed Tomography Small Bowel Feces Sign

**DOI:** 10.7759/cureus.114531

**Published:** 2026-08-14

**Authors:** Hayam S Almadani, Ahmed Alasmari, Mohammed Ali Alotaif, Mohammed Koumu, Khalid Bakri, Anhar Alqahtani, Ahmed Alhebi

**Affiliations:** 1 Department of General Surgery, Aseer Central Hospital, Abha, SAU; 2 Department of Bariatric and Upper Gastrointestinal Surgery, Aseer Central Hospital, Abha, SAU

**Keywords:** computed tomography, meckel's diverticulum, phytobezoar, small bowel feces sign, small bowel obstruction

## Abstract

Phytobezoar impaction within a Meckel's diverticulum is an exceptionally rare cause of mechanical small bowel obstruction and presents a diagnostic challenge because of its nonspecific clinical manifestations. We report the case of a 24-year-old previously healthy man who presented with a three-day history of progressive colicky abdominal pain, bilious vomiting, abdominal distension, and absolute constipation. Laboratory investigations revealed mild leukocytosis without evidence of systemic sepsis or biochemical evidence of bowel ischemia. Contrast-enhanced computed tomography demonstrated markedly dilated small bowel loops with an abrupt transition point in the distal ileum and a characteristic small bowel feces sign immediately proximal to the transition point, supporting the diagnosis and localization of mechanical obstruction. Emergency exploratory laparotomy identified an impacted phytobezoar within a Meckel's diverticulum causing complete luminal obstruction. No adhesions, congenital bands, volvulus, internal hernias, or gross bowel ischemia were identified. Segmental resection of approximately 30 cm of the distal ileum, including the Meckel's diverticulum and phytobezoar, followed by primary hand-sewn end-to-end anastomosis was successfully performed. Histopathological examination confirmed a Meckel's diverticulum with an impacted phytobezoar causing mechanical obstruction without transmural necrosis or established bowel ischemia. The patient had an uncomplicated postoperative recovery and remained asymptomatic during follow-up. This case highlights phytobezoar impaction within a Meckel's diverticulum as an uncommon cause of small bowel obstruction, particularly in young patients without previous abdominal surgery. It also illustrates the role of computed tomography in identifying and localizing mechanical obstruction and emphasizes the importance of timely surgical management when complete obstruction is present.

## Introduction

Small bowel obstruction is one of the most common surgical emergencies, accounting for approximately 12-16% of emergency surgical admissions and up to 20% of acute abdominal operations [1,2]. Although postoperative adhesions remain the leading cause in developed countries, accounting for nearly 60-75% of cases, the differential diagnosis is considerably broader in patients without a history of abdominal surgery, where congenital anomalies, neoplasms, inflammatory disorders, internal hernias, and bezoars should be considered [3]. Delayed diagnosis may result in bowel ischemia, perforation, sepsis, and increased postoperative morbidity. Therefore, prompt recognition of the underlying etiology is essential for timely surgical management.

Meckel's diverticulum is the most common congenital anomaly of the gastrointestinal tract, arising from incomplete obliteration of the omphalomesenteric duct [4]. It occurs in approximately 2% of the population, although only a minority of affected individuals develop complications during their lifetime [4]. In adults, intestinal obstruction is among the most important complications and may result from volvulus, intussusception, mesodiverticular bands, herniation, inflammatory strictures, or other mechanical mechanisms [5]. Phytobezoar impaction within a Meckel's diverticulum is an exceptionally uncommon mechanism of obstruction. Multiple individual case reports have been published over several decades, including recent reports and case series, but the available evidence remains limited to isolated cases and small series rather than systematic clinical studies [6-10].

Phytobezoars are concretions composed predominantly of indigestible vegetable and fruit fibers and represent the most common type of gastrointestinal bezoar [6]. They may occur in otherwise healthy individuals but are more frequently associated with factors such as previous gastric surgery, impaired gastric motility, and consumption of large amounts of high-fiber food [6]. On computed tomography (CT), a small bowel bezoar typically appears as a discrete intraluminal mass with a mottled appearance caused by intermixed gas bubbles and particulate material, often accompanied by proximal bowel dilatation [11]. This characteristic appearance represents the bezoar itself and should be distinguished from the small bowel feces sign, which describes fecal-like particulate material mixed with gas bubbles within dilated small bowel loops. The small bowel feces sign is a nonspecific secondary finding of intestinal stasis in mechanical small bowel obstruction and is most useful for helping identify or localize the transition point rather than determining the underlying cause [11,12]. In the present case, the small bowel feces sign was located immediately proximal to the transition point and supported the localization of the mechanical obstruction, whereas the diagnosis of phytobezoar impaction within the Meckel's diverticulum was established intraoperatively. We report a rare case of phytobezoar impaction within a Meckel's diverticulum causing complete distal small bowel obstruction, with particular emphasis on the clinical, operative, and histopathological findings and the role of CT in identifying and localizing the mechanical obstruction.

## Case presentation

A 24-year-old previously healthy man presented to the emergency department with a three-day history of progressively worsening colicky abdominal pain, accompanied by nausea, multiple episodes of bilious vomiting, progressive abdominal distension, and absolute constipation. He had no history of previous abdominal surgery, inflammatory bowel disease, chronic gastrointestinal disease, or regular medication use. He denied previous episodes of intestinal obstruction, gastrointestinal bleeding, fever, or weight loss. A detailed dietary history revealed no recent unusual dietary change, excessive consumption of fruits or vegetables, ingestion of large amounts of high-fiber or poorly digestible foods, or ingestion of indigestible material. This absence of an identifiable dietary precipitant was notable given the phytobezoar subsequently identified at surgery.

On admission, the patient was alert and hemodynamically stable. His vitals were as follows: temperature 37.2°C, blood pressure 118/76 mmHg, heart rate 88 beats/min, respiratory rate 16 breaths/min, and oxygen saturation 98% on room air. Physical examination revealed mild-to-moderate abdominal distension with generalized tenderness, more pronounced in the lower abdomen, without guarding or rebound tenderness. Bowel sounds were hypoactive, and no palpable abdominal masses or external hernias were identified. Digital rectal examination was unremarkable. Initial laboratory investigations demonstrated mild leukocytosis with a white blood cell count of 11.8×10⁹/L (reference range: 4.5-11.0×10⁹/L). Hemoglobin was 14.6 g/dL (reference range: 13.5-17.5 g/dL), platelet count 245×10⁹/L (reference range: 150-400×10⁹/L), serum lactate 1.2 mmol/L (reference range: 0.5-2.2 mmol/L), and C-reactive protein 12 mg/L (reference range: 0-5 mg/L). Serum electrolytes, renal function, and liver function tests were within normal limits, suggesting the absence of advanced bowel ischemia or systemic sepsis.

**Figure 1:**
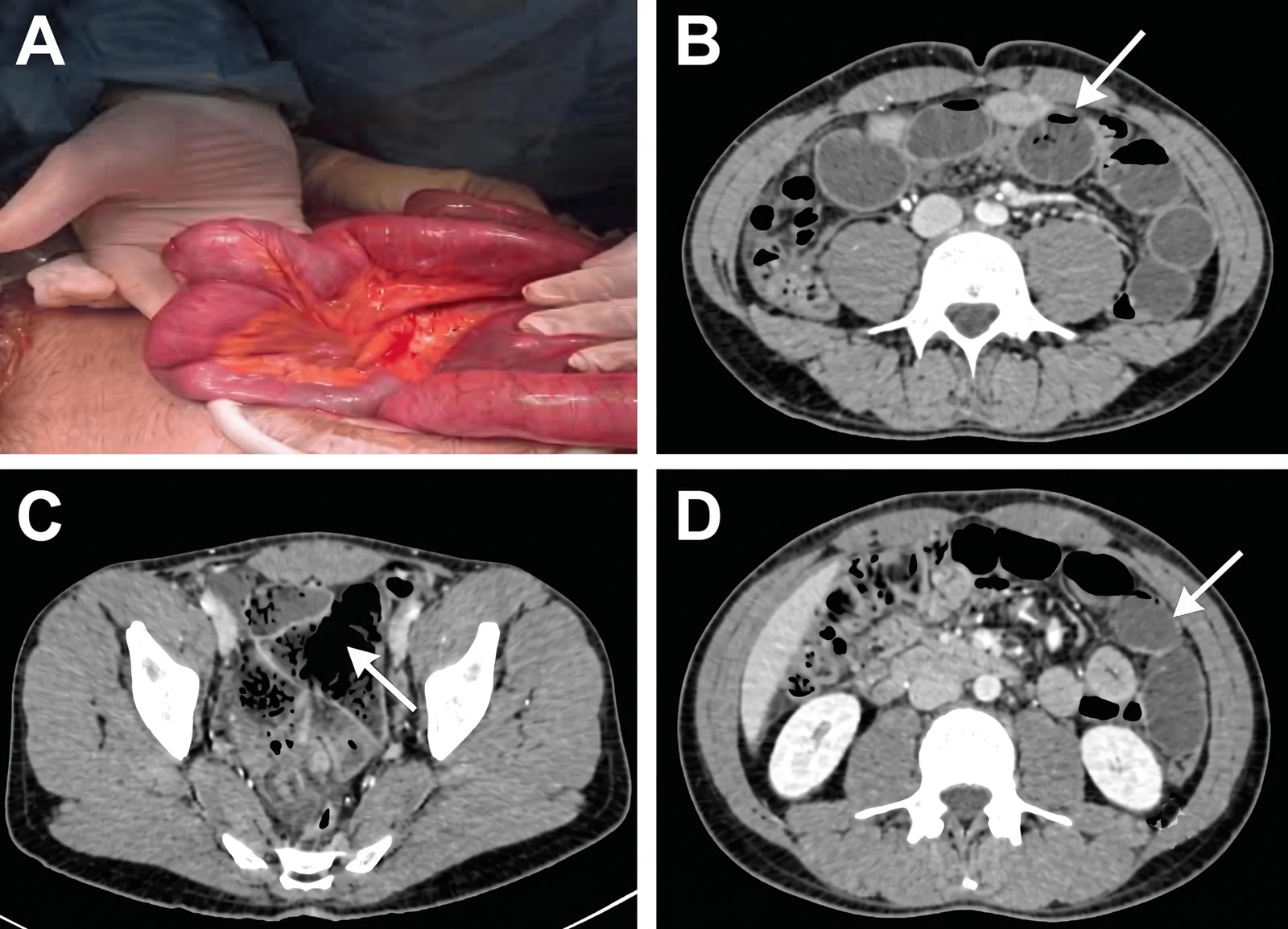
Radiological and intraoperative findings of phytobezoar-induced small bowel obstruction secondary to Meckel's diverticulum (A) Intraoperative photograph demonstrating dilated ileal loops at the site of obstruction caused by a phytobezoar impacted within a Meckel's diverticulum. (B) Axial contrast-enhanced CT image showing multiple dilated proximal small bowel loops (arrow) consistent with mechanical small bowel obstruction. (C) Axial CT image demonstrating the transition point in the distal ileum with the characteristic small bowel feces sign (arrow), represented by particulate intraluminal material intermixed with gas bubbles proximal to the obstruction. (D) Axial contrast-enhanced CT image demonstrating dilated proximal bowel loops (arrow) with decompressed distal bowel, confirming distal mechanical small bowel obstruction. CT: computed tomography

Contrast-enhanced CT of the abdomen and pelvis demonstrated multiple dilated proximal and mid-ileal loops measuring up to 4.2 cm in diameter, with an abrupt transition point in the distal ileum. Immediately proximal to the transition point, particulate intraluminal material intermixed with gas bubbles produced a characteristic small bowel feces sign, consistent with prolonged intestinal stasis. Mild mural thickening and adjacent mesenteric fat stranding were also present. No pneumoperitoneum, free intraperitoneal fluid, bowel wall pneumatosis, portal venous gas, or radiological evidence of bowel perforation or vascular compromise was identified. The findings were consistent with mechanical small bowel obstruction at the distal ileal level (Figure 1B-1D).

Following initial resuscitation with intravenous fluids, nasogastric decompression, analgesia, and prophylactic intravenous antibiotics, the patient underwent emergency exploratory laparotomy because of complete mechanical small bowel obstruction. Intraoperatively, markedly dilated proximal ileal loops were identified with an abrupt transition point at a Meckel's diverticulum in the distal ileum. The Meckel's diverticulum contained an impacted phytobezoar, which occupied the diverticular lumen and produced extrinsic compression and mechanical obstruction of the adjacent ileal lumen at the level of the diverticulum. Careful exploration of the abdominal cavity demonstrated no postoperative adhesions, congenital fibrous bands, internal hernias, volvulus, inflammatory strictures, or other mechanical causes of obstruction. The remaining small and large bowel appeared normal. Despite marked proximal dilatation, the obstructed bowel remained viable, with preserved color and mesenteric vascular pulsation and no gross evidence of ischemia, necrosis, or perforation (Figure 1A).

A segmental resection of approximately 30 cm of the distal ileum incorporating the Meckel's diverticulum, impacted phytobezoar, and adjacent obstructed ileal segment was performed, followed by primary end-to-end hand-sewn anastomosis. The resection was undertaken to definitively remove the obstructing Meckel's diverticulum and the adjacent segment involved in the obstruction and to permit the restoration of bowel continuity with viable resection margins. The extent of resection was not prompted by bowel ischemia, necrosis, or perforation, none of which was identified intraoperatively. The procedure was completed without intraoperative complications. Importantly, the indication for resection was the mechanical obstruction and anatomy of the involved segment rather than bowel ischemia, as no gross ischemia, necrosis, or perforation was identified.

The postoperative course was uneventful except for a small amount of superficial serous wound discharge. Cytological examination of two wound fluid specimens demonstrated benign inflammatory cells and lymphocytes without evidence of malignant or atypical cells. Bowel function returned uneventfully, oral intake was gradually resumed, and the patient was discharged home in good clinical condition after appropriate postoperative care. At follow-up, he remained asymptomatic, with normal bowel function and no evidence of recurrent bowel obstruction. A summary of the patient's clinical presentation, diagnostic evaluation, operative findings, and postoperative outcome is provided in Table 1.

**Table 1:** Summary of the patient's clinical course, diagnostic findings, management, and outcome WBC: white blood cell; CRP: C-reactive protein; CT: computed tomography; Ref: reference range

Domain	Findings
Patient characteristics	A 24-year-old previously healthy man with no previous abdominal surgery or chronic gastrointestinal disease
Presenting symptoms	Three-day history of progressive colicky abdominal pain, bilious vomiting, abdominal distension, and absolute constipation
Physical examination	Hemodynamically stable; mild-to-moderate abdominal distension; generalized lower abdominal tenderness; hypoactive bowel sounds; no peritoneal signs or external hernias
Laboratory findings	WBC 11.8×10⁹/L (Ref: 4.5-11.0×10⁹/L); hemoglobin 14.6 g/dL (Ref: 13.5-17.5 g/dL); platelet count 245×10⁹/L (Ref: 150-400×10⁹/L); CRP 12 mg/L (Ref: 0-5 mg/L); serum lactate 1.2 mmol/L (Ref: 0.5-2.2 mmol/L); normal renal function, liver function, and electrolytes
CT findings	Dilated proximal and mid-ileal loops (maximum diameter: 4.2 cm), abrupt distal ileal transition point, characteristic small bowel feces sign, mild mural thickening and mesenteric fat stranding, without pneumoperitoneum or bowel ischemia
Operative findings	Meckel's diverticulum containing an impacted phytobezoar causing complete luminal obstruction; no adhesions, congenital bands, volvulus, internal hernia, or bowel ischemia
Surgical procedure	Segmental resection of approximately 30 cm of the distal ileum including the Meckel's diverticulum with primary end-to-end hand-sewn anastomosis
Histopathology	Meckel's diverticulum containing an impacted phytobezoar causing complete mechanical obstruction without transmural necrosis or established bowel ischemia; viable resection margins
Postoperative course	Uneventful recovery; gradual resumption of oral intake; discharged in good clinical condition; asymptomatic during follow-up

## Discussion

Meckel's diverticulum is the most common congenital anomaly of the gastrointestinal tract, occurring in approximately 2% of the population as a result of incomplete obliteration of the omphalomesenteric duct [4]. Although most individuals remain asymptomatic, approximately 4-6% develop complications, including gastrointestinal bleeding, diverticulitis, perforation, and intestinal obstruction [13]. In adults, bowel obstruction is the most frequent presentation and is typically caused by volvulus, intussusception, mesodiverticular bands, inflammatory strictures, or adhesions rather than intraluminal obstruction by a bezoar [14,15].

Phytobezoars are aggregates of indigestible vegetable and fruit fibers and represent the most common type of gastrointestinal bezoar [6]. While they usually form within the stomach and migrate distally, impaction within a Meckel's diverticulum is exceptionally rare, with only a limited number of cases reported in the English-language literature [14]. A comparison of previously published cases is presented in Table 2. The proposed mechanism involves prolonged stasis within the blind-ending diverticulum, allowing progressive accumulation of vegetable fibers until complete luminal obstruction develops [16]. Unlike the more common mechanisms of obstruction associated with Meckel's diverticulum, our patient had no adhesions, congenital bands, volvulus, internal hernia, or inflammatory strictures, confirming phytobezoar impaction as the sole cause of obstruction.

**Table 2:** Clinicopathological characteristics of published cases of phytobezoar-induced SBO secondary to Meckel's diverticulum SBO: small bowel obstruction; NR: not reported; M: male; F: female; CT: computed tomography

Study	Year	Country	Age/sex	Clinical presentation	CT findings	Small bowel feces sign	Operative findings	Surgical management	Outcome
Hamburger [17]	1960	USA	30/M	Acute intestinal obstruction	NR	NR	Meckel's diverticulum associated with phytobezoar causing obstruction	Open surgical treatment	Recovered
Lawrence [8]	1982	UK	23/M	Abdominal symptoms initially attributed to pelvic appendicitis	NR	NR	Meckel's diverticulum with coconut bezoar causing intestinal obstruction	Surgical resection	Recovered
Sorensen and Ghani [9]	1992	USA	NR	SBO	NR	NR	Phytobezoar obstructing Meckel's diverticulum	Surgical treatment	Recovered
Mares et al. [18], Case 1	1993	Israel	10/M	SBO	NR	NR	Phytobezoar lodged within Meckel's diverticulum in a Y-shaped "pantaloon" configuration	Surgical treatment	Recovered
Mares et al. [18], Case 2	1993	Israel	11/M	SBO	NR	NR	Phytobezoar lodged within Meckel's diverticulum in a Y-shaped "pantaloon" configuration	Surgical treatment	Recovered
Frazzini et al. [19]	1996	USA	NR	Acute SBO	CT demonstrated phytobezoar-related distal SBO and characteristic intraluminal material	NR	Phytobezoar within Meckel's diverticulum with extension into the ileal lumen	Segmental ileal resection	Recovered
Colombo et al. [20]	2009	Italy	65/M	Acute SBO	Dilated small bowel loops with air-fluid levels; CT supported obstruction	NR	Meckel's diverticulum containing phytobezoar	Segmental ileal resection	Recovered
Duman et al. [21]	2011	Türkiye	16 months/M	Intestinal obstruction	Ultrasound and radiography used; CT not reported	NR	Phytobezoar within Meckel's diverticulum	Open surgical treatment	Recovered
Fagenholz and de Moya [15]	2011	USA	34/M	Episodic abdominal pain and vomiting with SBO	CT demonstrated SBO with intraluminal solid material	NR	Impacted phytobezoar within Meckel's diverticulum	Laparoscopic resection/diverticulectomy with bezoar removal	Recovered
Bini et al. [14]	2012	Italy	50/M	Abdominal pain, nausea, and vomiting	Dilated small bowel loops with air-fluid levels	NR	Giant Meckel's diverticulum filled with phytobezoar	Segmental ileal resection	Recovered
Gupta et al. [22]	2012	India	NR	Acute intestinal obstruction	NR	NR	Phytobezoar impacted at the base of Meckel's diverticulum	Surgical removal/resection	Recovered
Bingham et al. [16]	2014	USA	29/M	SBO after excessive fruit ingestion	Distal ileal fecalization, bowel wall thickening, and mesenteric inflammation	Yes	Wide-mouthed Meckel's diverticulum containing phytobezoar	Segmental ileal resection	Recovered
Gasparella et al. [10]	2016	Italy	12/M	Bowel obstruction/peritonitis	Ultrasound; CT not reported	NR	Meckel's diverticulum containing phytobezoar	Open resection	Recovered
Hussein et al. [23]	2017	UAE	47/M	Abdominal pain, vomiting, and constipation	Imaging demonstrated mechanical SBO	NR	Phytobezoar impacted within Meckel's diverticulum	Surgical resection	Recovered
Tatekawa [24]	2018	Japan	11/M	Intestinal obstruction after citrus-fruit ingestion	CT demonstrated intestinal obstruction	NR	Food/phytobezoar-associated obstruction involving Meckel's diverticulum	Laparoscopic surgery	Recovered
Khan et al. [25]	2019	Pakistan	13/M	Abdominal pain, distension, and constipation	Ultrasound and radiography; CT not reported	NR	Phytobezoar at the origin/base of Meckel's diverticulum	Wedge resection with primary closure	Recovered
Firat et al. [26], Case 1	2022	Türkiye	34/F	Acute intestinal obstruction	X-ray and CT demonstrated SBO	NR	Phytobezoar within Meckel's diverticulum	Open surgery	Recovered
Firat et al. [26], Case 2	2022	Türkiye	NR	Acute intestinal obstruction	X-ray and CT demonstrated SBO	NR	Phytobezoar within Meckel's diverticulum	Open surgery	Recovered
Zello and Kirschner [27]	2023	Canada	8/M	SBO	X-ray, ultrasound, and CT	NR	Phytobezoar-associated obstruction within Meckel's diverticulum	Surgical treatment	Recovered
Hulf and Ben-David [28]	2023	Australia	40s/M	Vomiting and obstipation after ingestion of large quantities of mango	CT demonstrated SBO with transition point	NR	Mango phytobezoar at Meckel's diverticulum producing intraluminal obstruction	Small bowel resection with primary anastomosis	Recovered
Ng et al. [29]	2023	Australia	21/F	Colicky abdominal pain, nausea, vomiting, and SBO	Distal SBO initially suspected to represent internal hernia	NR	Meckel's diverticulum containing phytobezoar; no obstructing fibrous band	Segmental ileal resection with anastomosis	Recovered
Soh et al. [30]	2024	Senegal	22/M	Three-day history of bowel obstruction	Hyperdense circular structure, small bowel dilatation, and free fluid	NR	Calcified phytobezoar within Meckel's diverticulum	Diverticulectomy	Recovered
Present case	2026	Saudi Arabia	24/M	Three-day history of progressive colicky abdominal pain, bilious vomiting, abdominal distension, and absolute constipation	Dilated small bowel loops up to 4.2 cm with distal ileal transition point and particulate intraluminal material with gas bubbles	Yes	Impacted phytobezoar within Meckel's diverticulum causing complete obstruction; no adhesions, congenital bands, volvulus, internal hernia, ischemia, or perforation	Approximately 30 cm ileal resection with primary end-to-end hand-sewn anastomosis	Recovered

The clinical presentation of phytobezoar-induced small bowel obstruction is nonspecific and resembles other causes of mechanical obstruction, making preoperative diagnosis challenging [7,16]. Typical CT findings include a well-defined intraluminal mottled mass containing gas bubbles with proximal bowel dilatation [12,31]. In our patient, the characteristic small bowel feces sign was present immediately proximal to the transition point. Although not specific for bezoars or Meckel's diverticulum, this sign reflects prolonged intestinal stasis and serves as an important radiological indicator of mechanical obstruction requiring prompt surgical intervention [11].

The differential diagnosis of mechanical small bowel obstruction in a patient without previous abdominal surgery is broad. Adhesive obstruction was initially less likely given the absence of prior abdominal surgery, although adhesions cannot be excluded reliably on CT alone; operative exploration subsequently demonstrated no adhesions. Internal hernia was considered because of the distal transition point and clustered dilated small bowel loops, but CT showed no convincing closed-loop configuration, abnormal mesenteric vessel arrangement, or herniation, and no internal hernia was identified at surgery. Crohn's disease was considered less likely because the patient had no previous inflammatory bowel disease or chronic gastrointestinal symptoms and CT did not demonstrate the characteristic combination of segmental inflammatory thickening, skip lesions, or fistulizing disease. Intussusception was not supported by the CT appearance, which showed no bowel-within-bowel configuration or lead-point mass. Gallstone ileus was unlikely because there was no ectopic gallstone, pneumobilia, or evidence of a biliary-enteric fistula. Similarly, a small bowel neoplasm was not identified as a discrete enhancing or obstructing mass on CT. Thus, although CT established the presence and distal ileal level of mechanical obstruction, the unusual etiology was not definitively established preoperatively and was confirmed only during surgical exploration [19]. This diagnostic uncertainty is consistent with the broad range of intrinsic, extrinsic, and intraluminal causes that may produce similar CT manifestations of small bowel obstruction [19].

Management depends on bowel viability and the underlying pathology. Conservative treatment has a limited role in complete mechanical obstruction, and surgery remains the definitive treatment [11]. Reported surgical options include enterotomy with bezoar extraction, fragmentation and milking of the bezoar into the colon, diverticulectomy, or segmental ileal resection [7,32,33]. When obstruction arises from a Meckel's diverticulum, segmental ileal resection with primary anastomosis is generally preferred because it removes both the obstructing bezoar and the underlying pathological segment [14,15]. In our patient, early surgical intervention resulted in complete resolution of the obstruction without bowel ischemia or postoperative complications.

This case expands the limited literature on phytobezoar impaction within a Meckel's diverticulum and highlights several important clinical lessons. Phytobezoar should be considered in the differential diagnosis of small bowel obstruction in patients without previous abdominal surgery, particularly when CT demonstrates a transition point accompanied by the small bowel feces sign. Early recognition and timely surgical management are essential to prevent bowel ischemia and achieve favorable outcomes.

Limitations

This report has several limitations. As a single case report, it cannot establish causal relationships or provide findings that are broadly generalizable to patients with Meckel's diverticulum or phytobezoar-induced small bowel obstruction. The rarity of this condition also limits comparison with alternative diagnostic and surgical approaches. In addition, the duration of follow-up was limited; therefore, long-term outcomes, particularly the risk of recurrent obstruction, cannot be fully assessed. Finally, although the small bowel feces sign supported the diagnosis and localization of mechanical obstruction, it is a nonspecific finding of intestinal stasis and should not be considered specific for phytobezoar impaction or Meckel's diverticulum.

## Conclusions

Phytobezoar impaction within a Meckel's diverticulum is an exceptionally rare cause of mechanical small bowel obstruction and may be difficult to diagnose preoperatively because its clinical presentation is nonspecific. Contrast-enhanced computed tomography is valuable for confirming and localizing mechanical obstruction, while the small bowel feces sign should be interpreted as a nonspecific marker of intestinal stasis rather than as evidence of a specific underlying cause. In young patients presenting with small bowel obstruction and no history of previous abdominal surgery, uncommon causes such as phytobezoar impaction within a Meckel's diverticulum should be considered when common etiologies are not apparent. In cases of complete mechanical obstruction, timely surgical exploration can provide definitive diagnosis and treatment.
