# Correction: Phytobezoar Impaction Within Meckel's Diverticulum Causing Distal Small Bowel Obstruction: A Case Report With Characteristic Computed Tomography Small Bowel Feces Sign

**DOI:** 10.7759/cureus.c474

**Published:** 2026-08-26

**Authors:** Hayam S Almadani, Ahmed Alasmari, Mohammed Ali Alotaif, Mohammed Koumu, Khalid Bakri, Anhar Alqahtani, Ahmed Alhebi

**Affiliations:** 1 Department of General Surgery, Aseer Central Hospital, Abha, SAU; 2 Department of Bariatric and Upper Gastrointestinal Surgery, Aseer Central Hospital, Abha, SAU

This article has been corrected to add Mohammed Ali Alotaif as third author after he was accidentally omitted from the initial publication.

